# Reply to “Comparative evaluation of the microbicidal activity of low-temperature sterilization technologies to steam sterilization”

**DOI:** 10.1017/ice.2020.122

**Published:** 2020-08

**Authors:** Randal Eveland

**Affiliations:** Steris, Mentor, Ohio

*To the Editor—*This letter is in response to the article by Rutala et al^1^ that compared the microbial kill of steam, ethylene oxide (ETO), hydrogen peroxide gas plasma (HPGP), and vaporized hydrogen peroxide (VHP) in the presence of salt and serum in standard sterilization cycles.

Unfortunately, at this time, there are no ‘standard’ gaseous hydrogen peroxide sterilization processes. The article fails to consider that although both HPGP and VHP processes use gaseous hydrogen peroxide as the sterilant, the processes are distinct and different in the way they operate. Even though 28-minute HPGP and VHP cycles are used, these cycles use significantly different concentrations of sterilant. The HPGP exposure is 25.6 mg/L H_2_O_2_ for 7 minutes whereas the VHP exposure is 9.1 mg/L H_2_O_2_ for 12 minutes. The importance of disinfectant concentration is explained in the 2008 CDC Guideline for Disinfection and Sterilization in Healthcare Facilities where it is stated that “The more concentrated the disinfectant, the greater is its efficacy and the shorter the time necessary to achieve microbial kill.”^2^ For these evaluations with no chamber load, sterilant concentration should have been considered.

The delineation of the gaseous hydrogen peroxide processes like HPGP and VHP, with the subsequent comparisons of efficacy minus any consideration of sterilant concentration, seems to imply that there is a benefit from plasma within the sterilization process. This contention contradicts the current understanding of the purpose of a gas plasma in HPGP systems, in which it is known that the plasma step has little to no contribution to sterilizer efficacy. In the only research ever published to evaluate the impact of plasma in a HPGP process, the plasma phase appeared to be nonsporicidal.^3^

The detoxifying (residual sterilant removing) effect of the plasma would have no impact on gaseous hydrogen peroxide microbial lethality; thus, the ~3-fold sterilant concentration difference (25.6 vs 9.1 mg/L H_2_O_2_ for the HPGP and VHP systems, respectively) is clearly responsible for the observed efficacy differences in HPGP and VHP processes. Higher concentration is not always beneficial. Beyond efficacy, hospitals also consider the gentleness of the sterilization process to include the potential impact of higher sterilant concentrations and higher sterilant dose on device material compatibility (especially devices susceptible to reaction with the highly oxidizing hydrogen peroxide sterilant) or device biocompatibility as well as the potential impact of plasma on medical device surfaces.

Both the HPGP and VHP sterilization cycles have been cleared by the Food and Drug Administration (FDA), so both have demonstrated the ability to achieve a sterility assurance level (SAL) of 10E-6 for their claimed processes. The CDC disinfection guidelines^2^ specify that even salts dissolved within surrogate body fluids dissolve with 60 seconds of nonflowing water; therefore, showing that, from a use perspective, the protective nature of salt has little clinical relevance. Although salt has been shown historically by many investigators to potentially impede hospital sterilization of medical devices, the emphasis of these results should be to highlight the need for thorough cleaning methodologies.
